# Contextual and own-age effects in age perception

**DOI:** 10.1007/s00221-022-06411-w

**Published:** 2022-08-19

**Authors:** Karin S. Pilz, Hao Lou

**Affiliations:** 1 Department of Experimental Psychology, University of Groningen, Groningen, The Netherlands; 2 Cito Institute for Educational Measurement, Arnhem, The Netherlands

**Keywords:** Age, Face perception, Own-age bias, Contextual effects, Perceptual averaging

## Abstract

Our judgement of certain facial characteristics such as emotion, attractiveness or age, is affected by context. Faces that are flanked by younger faces, for example, are perceived as being younger, whereas faces flanked by older faces are perceived as being older. Here, we investigated whether contextual effects in age perception are moderated by own age effects. On each trial, a target face was presented on the screen, which was flanked by two faces. Flanker faces were either identical to the target face, were 10 years younger or 10 years older than the target face. We asked 40 older (64–69 years) and 43 younger adults (24–29) to estimate the age of the target face. Our results replicated previous studies and showed that context affects age estimation of faces flanked by target faces of different ages. These context effects were more pronounced for younger compared to older flankers but present across both tested age groups. An own-age advantage was observed for older adults for unflanked faces who had larger estimation errors for younger faces compared to older faces and younger adults. Flanker effects, however, were not moderated by own-age effects. It is likely that the increased effect of younger flankers is due to mechanisms related to perceptual averaging.

## Introduction

Estimating someone’s age is an important aspect of our daily lives. We regularly, and often unconsciously, estimate the age of strangers or new acquaintances. Next to gender, and height, age is also one of the most common attributes used to describe a person. Also for legal reasons, for example, when assessing whether a customer is old enough to buy age-restricted products such as alcohol or tobacco, it is essential to be able to estimate someone’s age. Whereas research has shown that overall, participants are relatively good at estimating the age of a person (Awad et al. 2020; Henss 1991; Rhodes 2009), there are studies highlighting that the age of the depicted person matters. A common phenomenon here is that the age of teenagers and younger adults is often overestimated (Rhodes 2009; Rowe 2001; Vestlund et al. 2009; Voelkle et al. 2012), a phenomenon with huge implications for the sale of age-restricted products.

In addition to the overestimation of young faces, also an own-age advantage (sometimes referred to as own-age bias) has been observed in age estimations, such that participants are usually best at estimating the age of faces from within their own age group (Moyse and Brédart 2012; Voelkle et al. 2012). This effect exists across all ages, even though overall, older adults’ ability to estimate the age of faces generally decreases, and younger faces are overall judged more accurately than older faces.

A more recent study has found that age estimations do not only depend on the age of the observer and that of the face to be judged, but also on the context in which a face is presented (Awad et al. 2020). Contextual effects in visual perception are well known across a variety of different domains. An eminent illusion related to this context dependency is the Ebbinghaus illusion, in which two equally sized circles are placed close to each other, one surrounded by larger circles, the other one surrounded by smaller circles. Surprisingly, the central circle surrounded by larger circle appears smaller, whereas the central circle surrounded by smaller circles appears larger (Ebbinghaus 1902; Titchener 1901). Similar effects have been found for contrast and luminance (Lavrenteva and Murakami 2018; White 1981), orientation (Gibson 1937; Lavrenteva and Murakami 2018) and motion. These contextual effects on low-level visual features have been suggested to aid the perception of textures and scenes (Whitney and Yamanashi Leib 2018). Contextual effects have also been observed for higher level stimuli such as faces, such that the judgement of a particular aspect of a target face is affected by the surrounding faces. Facial characteristics for which contextual effects have been observed include emotion (Haberman and Whitney 2009), attractiveness (Walker and Vul 2014), gender (Haberman and Whitney 2009), and identity (de Fockert and Wolfenstein 2009; Neumann et al. 2013). Interestingly, these higher level contextual effects are assimilative rather than repulsive as the low-level contextual effects described above. It is thought that when contextual effects are assimilative, it is a result of averaging, such that the target face appears more similar to the context than it physically is, an effect that increases with increasing similarity between target and flanker faces (Awad et al. 2020; Whitney and Yamanashi Leib 2018).

Emotion or attractiveness are qualitative facial characteristics and therefore, difficult to measure. To be able to measure contextual effects quantitatively, Awad et al. (2020) used age as the variable of interest. They asked participants to judge the age of faces ranging from 15 to 65 years. The face was either viewed on its own, or was flanked by faces that were either both younger or both older than the target face. Results showed that faces that were flanked by younger faces were estimated to be younger, whereas faces flanked by older faces were estimated to be older compared to their veridical age. Interestingly, the effect of younger flankers was more pronounced than the effect of older flankers. These results could be due to an interplay of two phenomena: perceptual averaging and assimilation. Perceptual averaging is a phenomenon that allows us to quickly perceive the gist of a scene by averaging across scene content. In the case of flankers, it is possible that small blemishes or wrinkles are smoothed, decreasing the perceived age of the target faces. Assimilation refers to the effect that the age of a target face appears similar to the age of the flankers. Awad et al. suggest that these two phenomena act in the same direction in the case of younger flankers, but in opposite directions when the flankers are older, therefore, balancing each other out and resulting in smaller response errors.

Awad et al. (2020) only tested younger participants up to the age of 35 years and suggest that the increased effect of younger flankers is due to an own-age bias, such that younger participants pay more attention to younger flankers. However, they tested target faces between 15 and 65 years, and it is unlikely, that such an own-age bias moderated flanker effects even for older target faces. In addition, older flankers at the younger ages tested would be closer to the participant age than the younger flankers. Therefore, it is unlikely that an own-age bias explains the increased effect of younger flankers across the whole age range tested.

To further investigate the mechanisms underlying contextual effects in age estimation and to assess whether flanker effects are modulated by own-age effects, we tested 40 older and 43 younger participants in a task similar to the one introduced by Awad et al. (2020). Our hypothesis is threefold. First, we expect to replicate the results found by Awad et al. (2020) such that flankers affect age estimations of target faces across all target ages and participant age groups with an increased effect of younger compared to older flankers. Second, we expect to find an own-age advantage, such that older adults are better at estimating the age of older faces and younger adults are better at estimating the age of younger faces, in particular for unflanked faces. Third, it is reasonable to assume that the effect of flankers on age estimations is more pronounced when the flankers’ age can be estimated correctly. Therefore, we expect flanker effects for older adults to be more pronounced for older faces and flanker effects for younger adults to be more pronounced for younger faces.

## Methods

### Participants

Forty-three younger (23–29 years, *M* = 25.6, SD = 1.8) and 40 older participants (64–69 years, *M* = 65.5, SD = 1.4) took part in this study. Participants were recruited via Prolific and received 2.80 GBP for their participation. All participants were Caucasian, had self-reported normal or corrected-to-normal visual acuity, and reported no signs of mild cognitive impairment. The experiment was approved by the Ethics Committee of Psychology at the University of Groningen. All participants gave written informed consent.

Four younger and one older participant were excluded from the analysis, because their performance exceeded 2.5 SD away from the group mean in at least two conditions.

### Stimuli

Stimuli came from two databases, the Academic MORPH database and the Park Aging Mind Laboratory UTDallas face database (Minear and Park 2004). All images were Caucasian neutral faces, transformed to black and white and cropped to 300 by 450 pixels. Images with memorable facial characteristics such as scars or moles, open mouths or accessories were excluded. All faces were forward facing with their eyes angled directly at the camera.

### Pilot Study

**Fig. 1:**
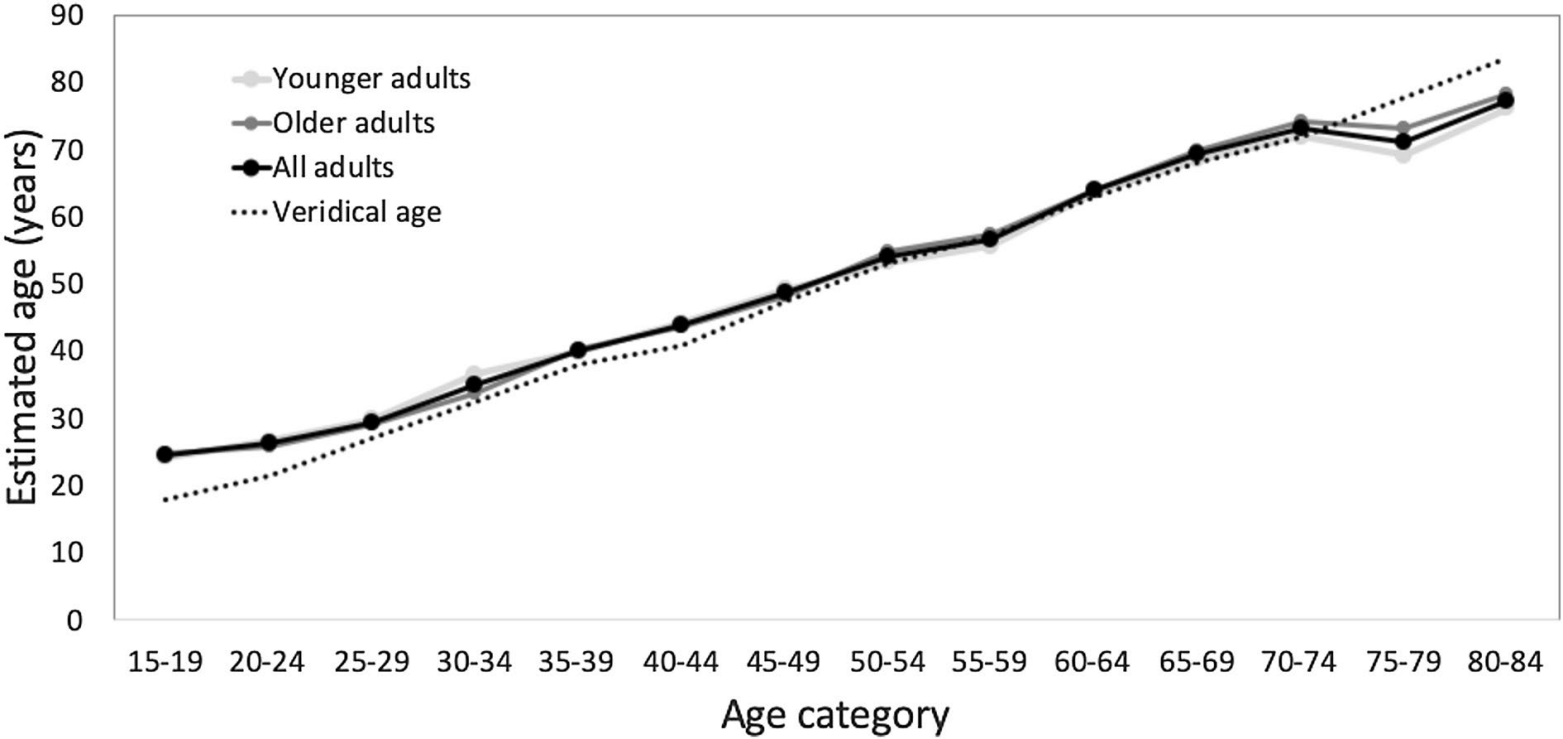
Age estimations for the faces used in this study from the pilot study. Younger faces are slightly overestimated and older faces are slightly underestimated. There is no significant difference between older and younger adults

To select the faces that were included in our experiment, we ran a pilot study in 20 older (10 male, 60–72 years, *M* = 65.1, SD = 4.1) and 20 younger participants (11 male, 20–34 years, *M* = 27.5, SD = 3.2) to identify those faces whose perceived age most closely matched their veridical age (closest mean estimates and smallest standard deviation). The physical age of the images varied from 16 to 92 years. Faces were sorted into age ranges of 5 years from > 20 to < 85 years old, and participants were asked to assess the age range of the faces. We estimated, which faces had the closest mean estimates and smallest standard deviation to their respective age range. This ensured that in our main experiments, the flankers were likely to be perceived as younger/older than the target. Interestingly, there was no significant difference in ratings between age-groups. The final set of faces that was used within this study consisted of 60 faces (6 within each age range) with 29 female and 31 male faces (see Fig. 1). It is interesting to note that similar to previous studies, the age of younger faces is overestimated, whereas the age of older faces is underestimated (Awad et al. 2020; Rhodes 2009; Rowe 2001).

### Procedure

**Fig. 2:**
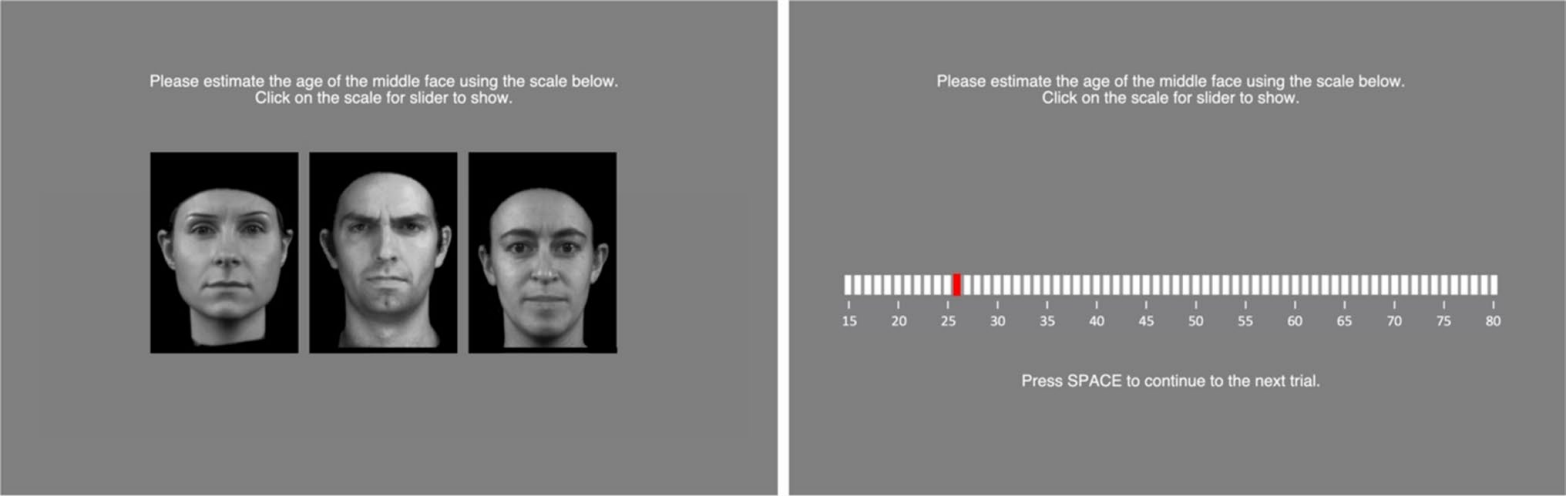
Example screenshots of the experiment. Participants saw three faces on the screen for 2000 ms and were asked to estimate the age of the middle face using a scale from 15 to 80 years that appeared once the faces had disappeared. After providing their answer, participants had to press the space bar to proceed to the next trial. Images shown are taken from the MPI moving face database for demonstration purposes only, and were not used in the current experiment (Pilz et al. 2006)

The experiment was run online using PsychoPy (Peirce et al. 2019) on Pavlovia. Stimuli were presented in the centre of the screen against a homogeneous grey background. Three flanker conditions were randomly interleaved. In the same flanker condition, the same face appeared three times on the screen. In the younger flanker condition, the target face was flanked by two faces that were in an age range of 10 years younger. In the older flanker condition, the target face was flanked by two faces that were in an age range of 10 years older than the target face (Fig. 2; images shown are for demonstration purposes only and were not used in the current experiment). A small gap of 5 pixels was added between the target and the flanking faces. Flankers were randomly chosen from the group of flanker faces from the appropriate age range. For each age range, there were six different target identities. Each target identity was shown once in each of the three flanker conditions, resulting in a total of 18 trials for each target age (six with younger flankers, six with older flankers and six with the same image as flankers), and 180 trials in total. Participants performed three blocks of trials and each target identity only appeared once per block of trials. Within each block, trials were randomised for each participant individually. Each trial began with a 500 ms fixation point, followed by a blank screen for 200 ms, followed by stimulus presentation for 2000 ms, followed by the response screen. Participants were asked to estimate the age of a target face using a scale ranging from 15 to 80 years (Fig. 2). Participants moved to the next trial by pressing the space bar. Participants were not informed about the age range of the stimuli and no feedback was given. The experiment took approximately 20 min to complete. Faces selected for the experiment were from people within the age ranges of 15–80 years. Given that flankers were always from an age range of 10 years older or younger than the target face, the range of target ages we were able to test was 25–74, resulting in 10 target age ranges (25–29, 30–34, 35–39, 40–44, 45–49, 50–54, 55–60, 60–64, 65–69, 70–74 years).

## Results

**Fig. 3:**
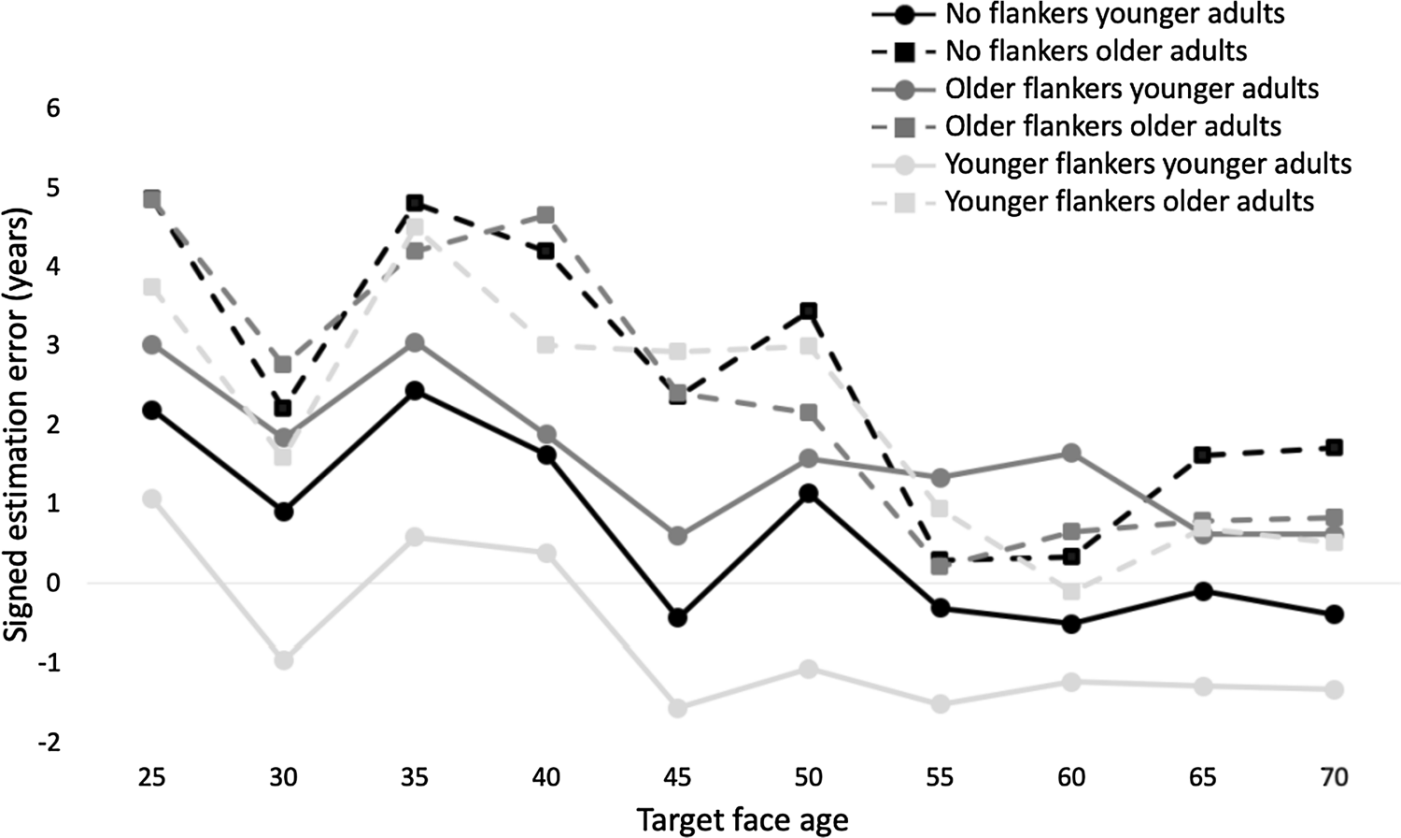
Signed estimation errors for younger (solid lines) and older adults (dashed lines) for no flankers (black), older flankers (dark grey) and younger flankers (light grey) for all target face age groups. Overall, older adults have larger estimation errors than younger adults, and the effect of flankers is less pronounced

Figure 3 shows signed estimation errors for older and younger adults for all three flanker conditions and all target face age groups. Interestingly, these estimation errors were overall largest for the youngest target face age and decreased with increasing target face age. For the analysis, we either collapsed across all target face ages to assess the overall effect of flankers (*The effect of flankers across target face age groups*), similar to Awad et al. (2020), or selectively assessed estimation errors for younger (target age range 25) or older faces (target age range 65) to assess own-age effects for faces and whether own-age effects moderate flanker effects (O*wn age effects for unflanked faces* and *Effects of flankers moderated by own-age effects*). All analyses were conducted with SPSS (2021).

### The effect of flankers across target face age groups

**Fig. 4:**
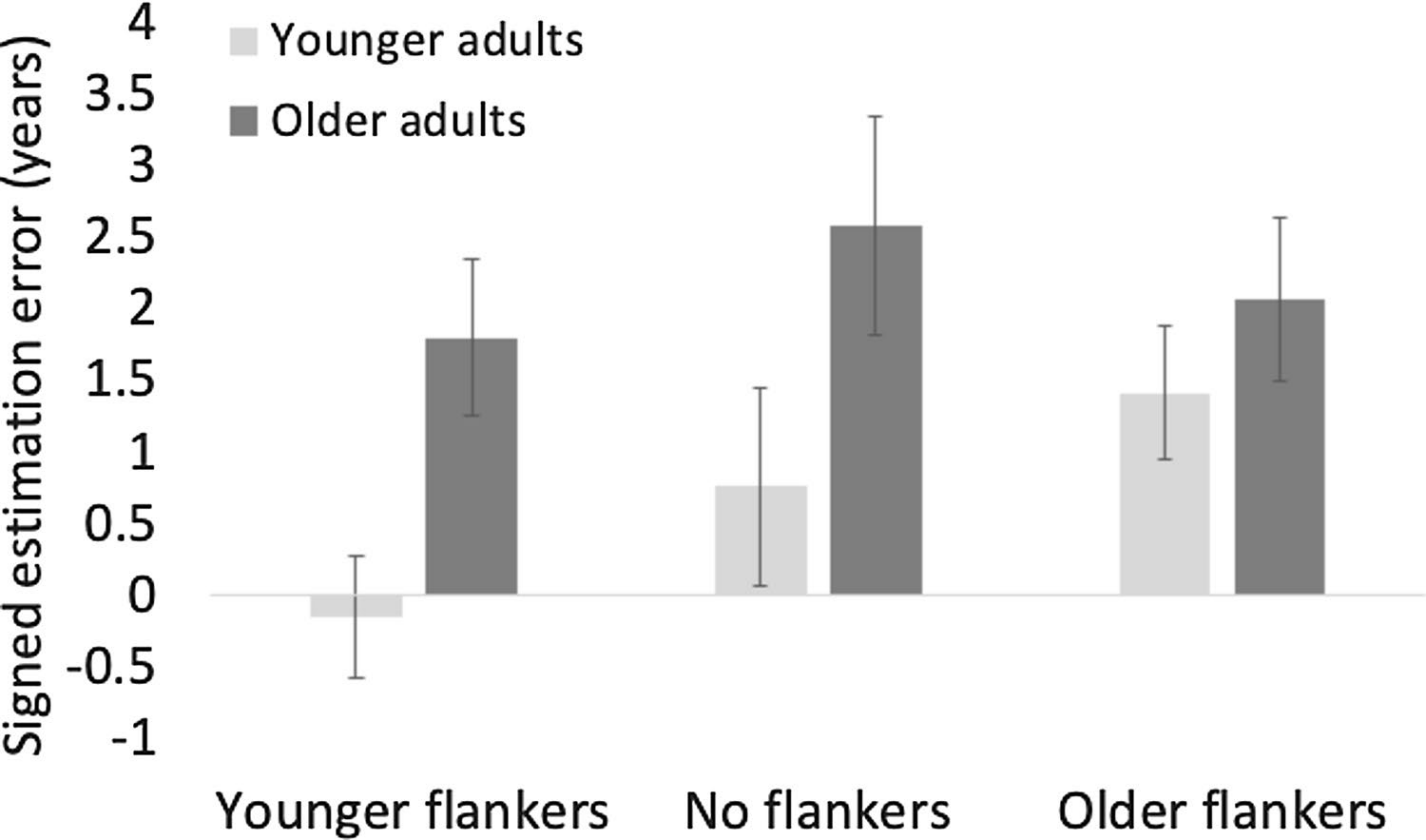
Signed estimation errors in years for older (dark grey) and younger adults (light grey) for all three flanker conditions collapsed across all target face ages ranging from 25 to 70 years. Error bars represent standard error from the mean. Older adults have overall larger estimation errors and the effect of flanker is more pronounced for younger adults

First, to assess the effect of flanker on age estimations across age groups, we ran a 2(age group) × 3(flanker type) mixed-design ANOVA on signed estimation errors with age group as a between subject factor (Fig. 4). Table 1 shows the descriptive values. Mauchly’s Test of Sphericity indicated that the assumption of sphericity had been violated. Therefore, Greenhouse–Geisser corrected degrees of freedom are reported. The ANOVA revealed a main effect of age group *F*(1,76) = 4.2, *p* < 0.05, ${\eta }_{p}^{2}$ =0.05, older adults had larger estimation errors than younger adults, and a main effect of flanker type, *F*(2,152) = 13.16, *p* < 0.001, ${\eta }_{p}^{2}$ =0.15, younger flankers significantly decreased age estimations compared to no flankers, *t*(76) = 4.7, *p* < 0.001, and to older flankers, *t*(76) = 3.6, *p* < 0.001, but there was no difference between older and no flankers, *t*(76) = 0.0, *p* = 0.28. The main effects were moderated by an age group x flanker type interaction, *F*(2,152) = 6.23, *p* < 0.01, ${\eta }_{p}^{2}$ =0.08. Post hoc tests showed that the effect of flanker was more pronounced for younger than older adults, with significant differences for younger adults between no flankers and younger flankers, *t*(76) = 3.4, *p* < 0.01, and older and younger flankers, *t*(76) = 5.89, *p* < 0.001. Older adults had significantly larger estimation errors than younger adults for younger flankers, *t*(38) = 2.7, *p* < 0.01, and for no flankers, *t*(38) = 2.2, *p* < 0.05. All other comparisons were not significant with *p* > 0.3 (Table 2).

**Table 1:** Means and standard deviations for older and younger adults for all three flanker types collapsed across face ages for signed estimation errors

Flanker	Age group	Mean	SD	*N*
No flanker	Older adults	2.311	3.582	39
–	Younger adults	0.754	2.425	39
older flanker	Older adults	2.066	3.591	39
–	Younger adults	1.409	2.909	39
younger flanker	Older adults	1.794	3.407	39
–	Younger adults	− 0.156	2.645	39

**Table 2:** Means and standard deviations (SD) for both age groups and both face ages for the no flanker condition for magnitude estimation errors

Face age	Age group	Mean	SD	*N*
Older faces	Older adults	4.015	2.692	39
–	Younger adults	3.222	2.114	39
Younger faces	Older adults	5.576	3.781	39
–	Younger adults	2.941	2.148	39

### Own age effects for unflanked faces

**Fig. 5:**
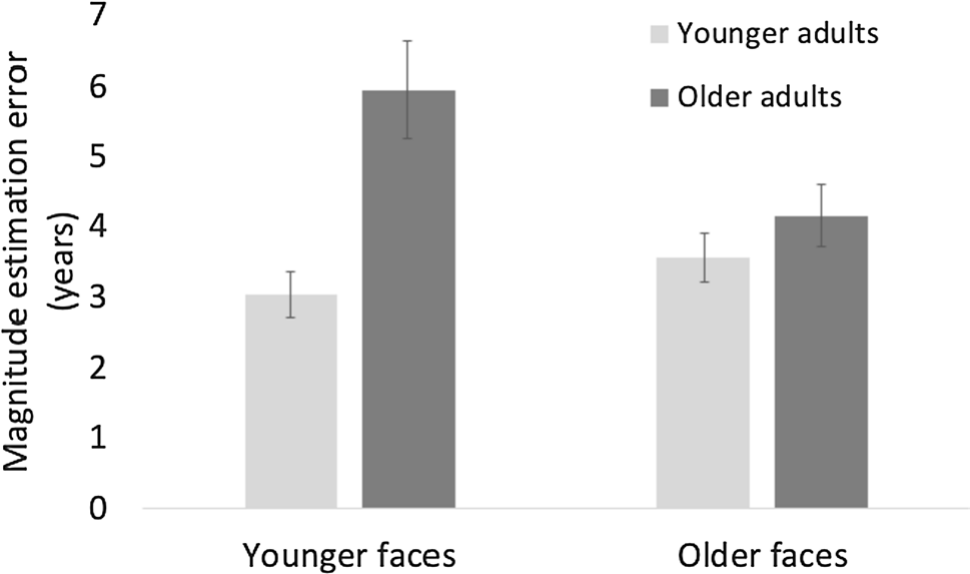
Magnitude estimation errors in years for unflanked target faces. Error bars represent standard errors from the mean. Older adults had overall larger estimation errors than younger adults. Further, the results confirm an own-age advantage for older adults who made larger errors for younger compared to older faces. The difference between age groups was more pronounced for younger faces

To assess own age effects and test whether older adults were better at estimating the age of older faces and younger adults were better at estimating the age of younger faces, we computed a 2(age group) × 2(face age) mixed-design ANOVA on the magnitude estimation error for the no flanker condition only (Fig. 5).

The ANOVA revealed a main effect of age group, *F*(1,76) = 15.52, *p* < 0.001, ${\eta }_{p}^{2}$ = 0.17, older adults had significantly larger errors than younger adults. The interaction face age × age group was also significant, *F*(1,76) = 4.2, *p* < 0.05, ${\eta }_{p}^{2}$ =0.05. There was no main effect of face age, *F*(1,76) = 2.02, *p* = 0.16.

Post hoc tests on the interaction between face age and age group revealed that older adults had significantly larger errors for younger faces than younger adults, *t*(76) = 3.88, *p* < 0.001.

In addition, older adults had significantly larger estimation errors for younger compared to older faces, *t*(38) = 2.5, *p* < 0.02. All other comparisons were not significant with *p* > 0.15.

### Effects of flankers moderated by own-age effects

**Fig. 6:**
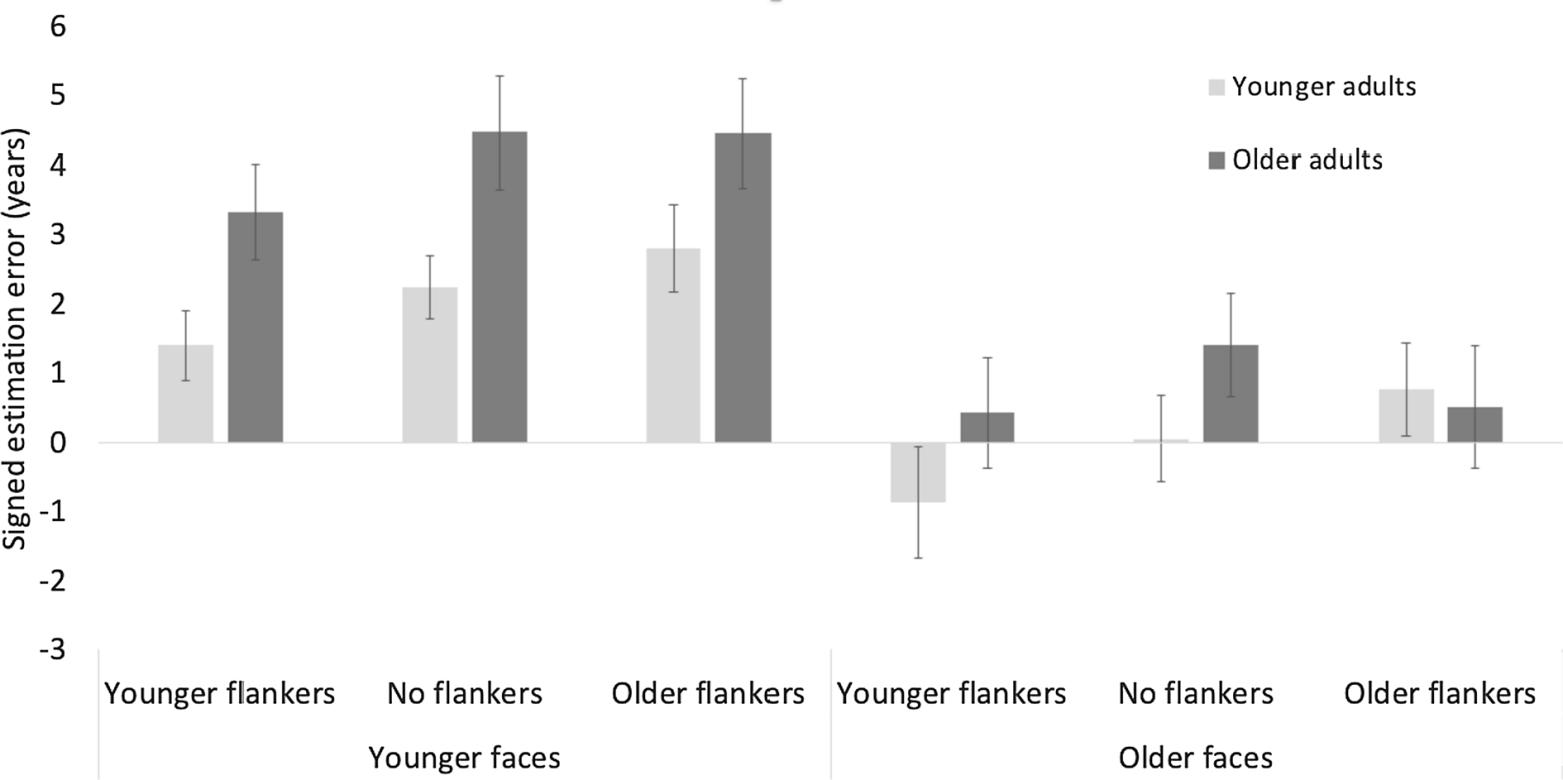
Signed estimation errors in years for older (dark grey) and younger adults (light grey) for all three flanker conditions for younger and older faces for. Error bars represent standard error from the mean. Estimation errors were larger for younger faces, which were overall overestimated. Older adults had overall larger estimation errors. Younger flankers significantly decreased age estimations, an effect that was more pronounced for older adults. The effect of older flankers was overall negligible

To assess the effect of flanker on age estimations and whether the effect was moderated by participant age and face age, we conducted a 2(age group) × 2(face age) × 3(flanker type) mixed-design ANOVA on the signed estimation error with age group as between-subject factor (Fig. 6). To be able to explicitly target own-age effects, we only included younger target faces (25 years) and older target faces (65 years) in this analysis. Table 3 shows descriptive statistics. Mauchly’s test indicated a violation of the assumption of sphericity. Therefore, Greenhouse Geisser corrected degrees of freedom are reported.

**Table 3:** Mean and standard deviation of signed estimation errors for younger and older adults for all three flanker conditions and face ages

Face age	Flanker type	Age group	Mean	SD	*N*
Older faces	No flanker	Older adults	1.405	4.665	39
–	–	Younger adults	0.045	3.889	39
–	Older flanker	Older adults	0.511	5.546	39
–	–	Younger adults	0.759	4.214	39
–	Younger flanker	Older adults	0.428	4.971	39
–	–	Younger adults	− 0.869	4.764	39
Younger faces	No flanker	Older adults	4.468	5.072	39
–	–	Younger adults	2.241	2.888	39
	Older flanker	Older adults	4.446	4.913	39
		Younger adults	2.804	3.927	39
–	Younger flanker	Older adults	3.327	4.350	39
–	–	Younger adults	1.398	3.163	39

**Fig. 7:**
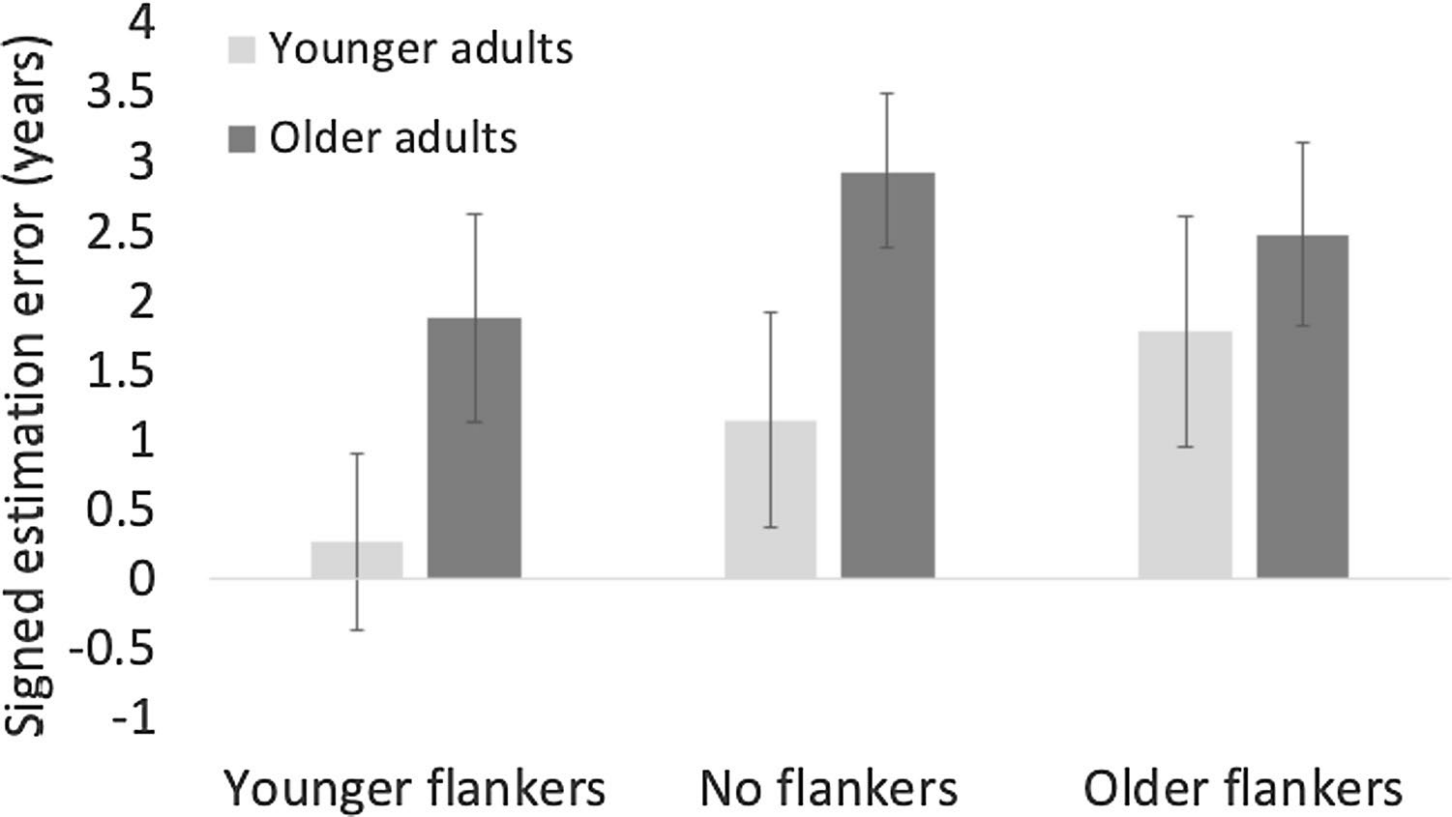
Signed estimation errors in years for younger (dark grey) and older adults (light grey) for all three flanker conditions collapsed across the two analysed face ages of younger (25 years) and older faces (65 years). Younger flankers significantly decreased age estimations, an effect that was more pronounced for older adults. The effect of older flankers was overall negligible

There was a main effect of age group, *F*(1,76) = 3.99, *p* < 0.05, ${\eta }_{p}^{2}$ =0.05, older adults had larger estimation errors than younger adults, a main effect of face age *F*(1,76) = 18.5, *p* < 0.001, ${\eta }_{p}^{2}$ = 0.2, the estimation error for younger faces was significantly larger than for older faces, and a main effect of flanker, *F*(2,152) = 12.68, *p* < 0.001, ${\eta }_{p}^{2}$ = 0.14, younger flankers significantly decreased age estimation compared to no flankers *t*(76) = 4.7, *p* < 0.001 and older flankers *t*(76) = 3.68, *p* < 0.001, whereas estimation errors for older flankers did not differ from those for no flankers *t*(76) = 0.3, *p* = 1. The main effects of age group, and flanker, were moderated by an age group x flanker interaction, *F*(2, 152) = 3.18, *p* = 0.05, ${\eta }_{p}^{2}$ =0.04. For older adults, post-hoc tests showed a significant difference between no flankers and younger flankers, *t*(38) = 3.3, *p* < 0.001. For younger adults, there was a significant difference between younger flankers and no flankers, *t*(38) = 3.14, *p* < 0.01, and a significant difference between older flankers and younger flankers, *t*(38) = 3.9, *p* < 0.001. There were also significant differences between older and younger adults for younger flankers, *t*(38) = 2, *p* < 0.05 and no flankers, *t*(38) = 2.6, *p* < 0.02, and estimation errors were larger for older adults. All other comparisons were not significant with ps > 0.12 (Fig. 7).

## Discussion

The estimation of age is an important ability, which we frequently use (mostly unconsciously) when encountering unfamiliar people. Here, we employed a flanker paradigm to investigate age estimations in context and to assess whether contextual effects in age estimations are moderated by participant and target age. Overall, we found contextual effects on age estimations such that younger flankers decreased age estimations compared to unflanked and older faces. The effect of younger flankers on age estimations was more pronounced for younger adults. Older flankers did not significantly affect age estimations compared to unflanked target faces. The own-age advantage for unflanked target faces was only partially fulfilled: older adults were overall worse at estimating the age of target faces compared to younger adults. However, they were better at estimating the age of older compared to younger faces and were worse at estimating the age of younger faces than younger adults. Our third analysis did not support a moderating effect of own-age on flanker effects, and mostly summarises the results from the previous two analyses such that overall, signed estimation errors were larger for younger than for older target faces, but target face age did not interact with participant age. Whereas older flankers only marginally affected age estimations for younger adults, younger flankers decreased age estimations for older and younger adults, an effect that was more pronounced for younger adults.

In accordance with previous studies, our results show that context affects the perception of target faces. Contextual effects have been found in many tasks, for many facial characteristics including emotion, gender, attractiveness and identity (de Fockert and Wolfenstein 2009; Haberman and Whitney 2009; Neumann et al. 2013; Walker and Vul 2014). Here, we extend results by Awad et al. (2020) and show that context also affects the estimation of age, irrespective of participant age. Even though, the effect of younger flankers was not as distinct for older adults as for younger adults, both age groups showed a decrease in age estimations when targets were flanked by younger faces. Interestingly, the effect of older flankers was not as pronounced as previously shown. However, it is important to note, that also Awad et al. (2020) found that younger flankers had a much larger effect on age estimations than older flankers. Two mechanisms have been proposed to explain the differential effect of older and younger flankers on age estimation: perceptual averaging and assimilation (Awad et al. 2020). Assimilation refers to the process that a target face simply appears similar to the flanker faces. However, if assimilation was underlying the results observed in our study, we would expect similar effects of older and younger flankers, unless assimilation is modulated by other phenomena such as own-age effects, for example. Perceptual averaging refers to our ability to efficiently process and perceive the gist of a scene, or the average value of a certain object feature or characteristic of a number of items presented at the same time. It could explain why we perceive an average emotion from a set of faces, or the average size of a set of objects presented with different sizes (Ariely 2001; Haberman and Whitney 2009). With regards to the effects of flankers on age estimations, it is likely that the effect of facial features that are relevant to estimate someone’s age, such as wrinkles or blemishes, is diminished during the process of averaging. A target face flanked by two younger faces, therefore, appears younger than the face presented on its own. If, however, a target face is flanked by two older flankers, it is possible that the same process reduces or annihilates the flanker effect. An idea to further investigate whether the differential effects of older and younger flankers on age estimation are due to perceptual averaging would be to increase the number of flankers presented. Based on the reasoning above, one would expect to find that increasing, for example, the number of younger flankers would further reduce the perceived age of the target.

Awad et al. (2020) propose another cause for increased effects of younger flankers, and suggest that, due to an own-age bias, younger participants pay more attention to younger compared to older flankers, which increases the influence of younger flankers in the perceptual average. Yet, in some conditions of their experiment, older flankers were closer to the participants’ age than younger flankers. Based on this argument, one would also expect to find that flankers in general have a larger effect on younger faces for younger adults, as the age of both older and younger flankers is closer to the participant age compared to younger flankers of middle-aged or older targets. Awad et al. (2020) only assessed flanker effects across target ages and, therefore, were unable to assess this hypothesis. However, our results suggest that flanker effects are not moderated by own-age effects.

Previous studies on face perception have found that participants are better at recognizing faces from within their own age group, thus, exhibiting an own-age bias (Anastasi and Rhodes 2005; Rhodes and Anastasi 2012). A similar bias has been observed for age estimations (Moyse and Brédart 2012; Short et al. 2019; Voelkle et al. 2012). Our results, however, only partially fulfil an own-age bias for unflanked faces. The main age difference in our study results from older adults being particularly bad at estimating the age of younger faces compared to older faces and compared to younger adults. These results are surprising on different levels. First, previous studies found that regardless of participant age, age estimations are better for younger than older target faces (Moyse and Brédart 2012; Short et al. 2019). Short et al. (2019) argue that due to an early exposure we receive with younger faces, the face processing system is selectively tuned to the recognition and assessment of younger adult faces, as we most frequently encounter younger adult faces during development (Rennels and Davis 2008). This early-acquired experience has been suggested to impact our ability to learn from encounters with new types of faces in adulthood (Macchi Cassia 2011).

However, our findings indicate that older adults are particularly bad at estimating the age of younger faces compared to younger adults and older faces. Second, it has been found that the own-age bias is often accompanied by a larger variability and increased errors for estimating the age of older faces (Moyse and Brédart 2012), which has been related to extrinsic factors such as smoking habits, sun exposure, nutrition or stress varying strongly between people (Nkengne et al. 2008; Rexbye et al. 2006). Though, our results indicate that overall, errors are more pronounced for younger faces.

It has to be noted that our study differs to previous studies on various levels, some of which can potentially explain discrepancies between our results and those of previous studies. First of all, our study was conducted online rather than in controlled experimental lab settings. As participants conducted the experiment on their own devices, we were unable to control for variables such as stimulus size or contrast. It is, for example, possible that older adults used overall larger devices. An increased stimulus size might have increased the effect of flankers for older adults. In addition, we were also unable to assess participants’ visual acuity beforehand, and had to trust that they would wear appropriate eyewear if necessary. Controlling for visual acuity and eye health is particularly relevant when testing older participants, and we cannot exclude the possibility that the general increase in errors for older adults is related to a decreased visual acuity or contrast sensitivity which is often found in ageing studies (Agnew et al. 2020; Agnew and Pilz 2017; Roudaia et al. 2010; Shaqiri et al. 2019).

There were also methodological differences between our study and previous studies. Whereas in our experiment, grayscale photographs were used as stimuli, most other studies assessing the own-age bias in age perception presented faces in colour (Awad et al. 2020; Moyse and Brédart 2012; Voelkle et al. 2012). This seemingly small difference could have important implications for the outcome of our study, as it has been shown that colour carries important information about facial age (Arce-Lopera et al. 2013; Burt and Perrett 1995; Matts et al. 2007; Puccetti et al. 2011; Russell et al. 2014). Manipulating the RGB colour intensity of faces, for example, has been found to affect age estimations (Burt and Perrett 1995), the redness or yellowness of the sclera of the eye has been related to the perceived age of a person (Russell et al. 2014), and the more colour saturated a face is, the older that face is perceived (Puccetti et al. 2011). Having used grayscale images in our study, therefore, might have deprived participants of necessary information to correctly judge the age of the presented faces. As a consequence, participants may have used different strategies than those usually adopted. Furthermore, it has been shown in previous studies that effects related to face recognition or perception often change with a particular stimulus set or image size used (Ross et al. 2015; Ross and Gauthie 2015). The fact that we were unable to control for stimulus size within our study, might have introduced additional noise and variability. In addition to using different stimuli, we also slightly changed the procedure to Awad et al., such that we presented the same target face three times for the no flanker condition. As in our study, all flanker and target conditions were randomised rather than blocked, we did this to ensure that participants were not confused by different stimulus presentations and knew to always estimate the age of the face presented in the middle.

In conclusion, we replicated previous studies and showed that context affects age estimation of faces flanked by target faces of different ages. Context effects were more pronounced for younger compared to older flankers but present across both tested age groups. It is likely that the increased effect of younger flankers is due to mechanisms relating to perceptual averaging.

## Data Availability

The datasets generated during and/or analysed during the current study are available from the corresponding author on reasonable request.
